# TCfinder: Robust tumor cell discrimination in scRNA‐seq based on gene pathway activity

**DOI:** 10.1002/imo2.22

**Published:** 2024-08-07

**Authors:** Chenxu Wu, Wei Ning, Tao Wu, Jing Chen, Huizi Yao, Ziyu Tao, Xiangyu Zhao, Kaixuan Diao, Jinyu Wang, Weiliang Wang, Xinxing Li, Qianqian Song, Xue‐Song Liu

**Affiliations:** ^1^ School of Life Science and Technology ShanghaiTech University Shanghai China; ^2^ Shanghai Institute of Biochemistry and Cell Biology, Chinese Academy of Sciences Shanghai China; ^3^ University of Chinese Academy of Sciences Beijing China; ^4^ Military Medical Innovation Center Air Force Medical University Xi'an China; ^5^ Department of Dermatology Yangjiang People's Hospital affiliated to Guangdong Medical University Yangjiang China; ^6^ Department of Colorectal Surgery Fudan University Shanghai Cancer Center Shanghai China; ^7^ Center for Cancer Genomics and Precision Oncology, Wake Forest Baptist Comprehensive Cancer Center Atrium Health Wake Forest Baptist Winston‐Salem North Carolina USA; ^8^ Department of Cancer Biology Wake Forest School of Medicine Winston‐Salem North Carolina USA; ^9^ Shanghai Clinical Research and Trial Center Shanghai China

## Abstract

TCfinder is a tumor cell identification tool, based on pathway activity and deep neural network (DNN). Across different platforms of scRNA‐seq datasets, TCfinder demonstrates robust identification efficiency. It outperforms existing tumor cell identification tools and performs under sparse data. TCfinder is freely available as an R package at: https://github.com/XSLiuLab/TCfinder.

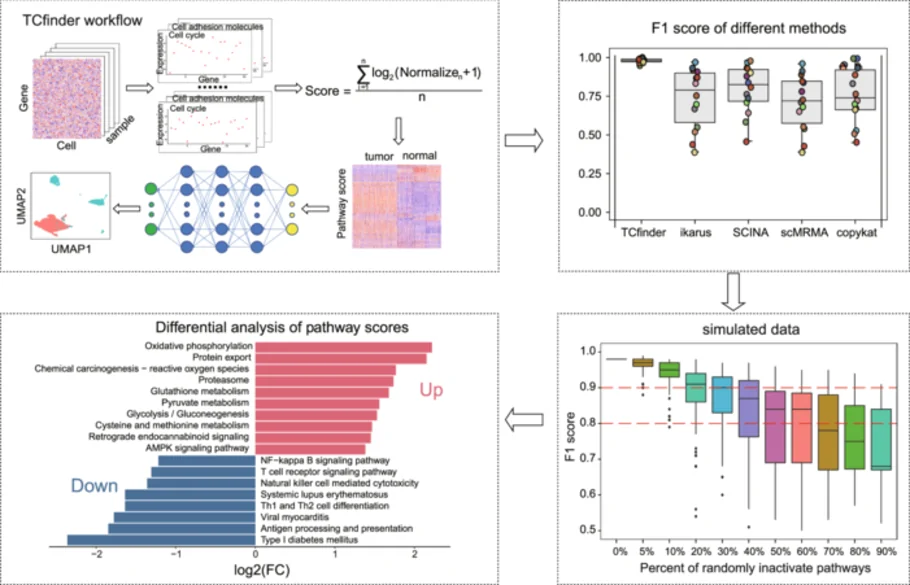

Traditional RNA sequencing (RNA‐seq) of bulk tumors only obtains the average information of gene expression [1], thus unable to precisely delineate the tumor microenvironment and infiltrating cell states. In contrast, single‐cell RNA sequencing (scRNA‐seq) technology allows to gain more in‐depth insights into the cellular ecosystem, including the interrogation of specific cell populations and their respective transcriptomic characteristics [2], the intratumoral heterogeneity [3], tumor evolution map [4], and tumor phylogenetic tree, and so on [5], which has been widely applied in cancer research.

One of the major challenges for cancer tissue‐related scRNA‐seq analysis is to accurately and quickly discriminate tumor cells from normal stromal cells [6]. Many cell‐type annotation methods have been constructed for scRNA‐seq analysis [7, 8]; however, few tools are available for tumor versus normal cell discrimination. Currently, there are two common strategies for distinguishing tumor cells from normal cells in scRNA‐seq data. One strategy involves clustering and manual annotation [9]. Although manual annotation can effectively distinguish tumor cells from normal cells in single‐cell data, it is still limited by the sparsity of single‐cell data sets and relies heavily on professional experience and knowledge, making it difficult to scale up. The other strategy involves automatic annotation, which includes methods based on marker genes, such as ikarus [10], SCINA [11], scMRMA [12], or methods based on copy number variation (CNV) inference [13]. However, this strategy is problematic due to extensive dropout issues in scRNA‐seq [14], resulting in insufficient expression of gene markers for most cancer cells. In addition, CNV is not universally prevalent in tumor cells, and some normal cells could have CNV [15], which limits the widespread application of CNV‐based methods. Therefore, it is of great importance to develop a widely applicable automatic annotation method that can overcome the sparsity of single‐cell data for the field of single‐cell‐based tumor research.

Since a typical gene pathway usually has dozens of genes, pathway‐based expression quantification overcomes data sparseness faced by traditional gene marker‐based annotating methods. Additionally, alterations in gene pathways are one of the primary differences between cancer cells and normal cells [16]. Therefore, characterizing gene pathways has great potential for accurately distinguishing between cancerous and normal cells.

Herein, we developed TCfinder (Tumor Cell finder) based on the pathway activity and deep neural network. TCfinder not only presents a robust performance in simulated scRNA‐seq data with random gene inactivation but also shows improved tumor versus normal cell discrimination precision and accuracy in multiple cancer types compared with existing methods.

## 1 COLLECTION OF PATHWAYS FOR TUMOR CELL DISCRIMINATION

We first analyzed the distribution of gene counts in tumor cells and normal cells (Figure S1). The number of genes measured in tumor cells is higher than in normal cells, which may be the result of more vigorous metabolism and growth of tumor cells. We collected all human gene pathways in the Kyoto Encyclopedia of Genes and Genomes database and scored the activities of each pathway (Methods for detail) to obtain a single‐cell pathway score matrix. In the training data set, we performed Wilcoxon tests on the pathway scores between tumor cells and normal cells, retaining pathways with *p* values <0.05. Ultimately, we identified 213 pathways to be used in TCfinder (Table S1).

**Figure 1:**
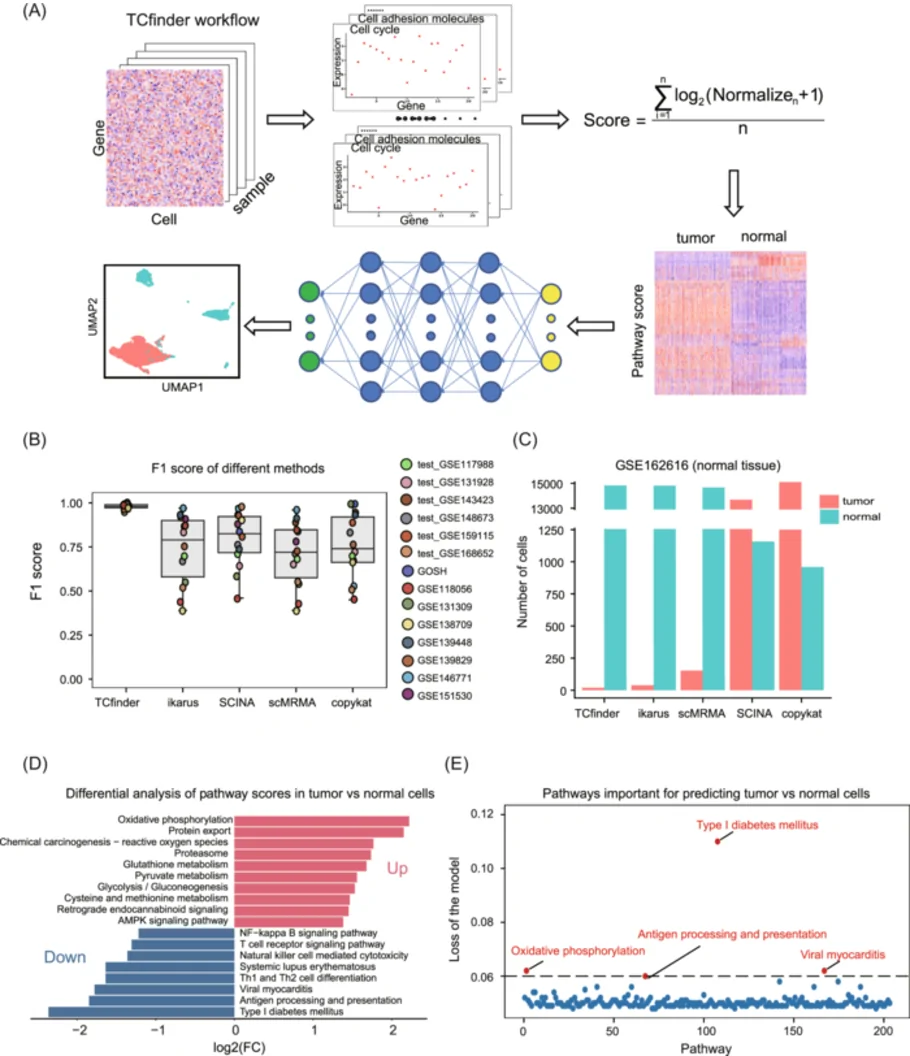
Performance of TCfinder in Identifying tumor cells. (A) TCfinder workflow. TCfinder is a gene pathway activity score‐based deep learning method for tumor cell discrimination. (B) Tumor versus normal cells classification performance of TCfinder and other known methods (ikarus, SCINA, copykat, and scMRMA). F1 score, accuracy, recall, and precision for each method in independent validation data sets are shown. (C) Performances of different methods (TCfinder, ikraus, SCINA, and scMRMA) in the GSE162616 (healthy tissue) data set are shown. (D) Differential analysis of pathway scores between tumor cells and normal cells showed pathways with |log2FoldChange| >1 and false discovery rate <0.05. (E) Pathways that contribute significantly to the classification of tumor and normal cells. The four most important pathways, type I diabetes mellitus, oxidative phosphorylation, viral myocarditis, and antigen processing and presentation, are shown.

In TCfinder, we utilized a fully connected neural network architecture to develop a model for discriminating between tumor and normal cells (Figure 1A). To develop a model broadly applicable at the pan‐cancer level, we collected over 70,000 cells from six types of cancer as training data. These data sets were randomly divided into training and test sets in an 8:2 ratio. Additionally, we used 10 data sets comprising over 230,000 cells as independent validation data for the model (Table S2).

## 2 COMPARING TCFINDER WITH EXISTING CELL ANNOTATION METHODS

The performance of TCfinder and existing methods, including ikarus, SCINA, copykat, scMRMA, was compared using independent data sets. TCfinder obtained an average F1 score of 0.98 on these data sets of the 10X platform and 0.95 on the SMART‐Seq2 platform (Table S3). The other four methods showed poorer performance, as reflected by lower F1 score, accuracy, and precision compared with TCfinder (Figures 1B and S2). Tumor versus normal status professionally annotated scRNA‐seq data sets are pretty limited. One possible strategy to address this issue is to use healthy tissue samples as normal samples and tumor cell line samples as tumor samples. To determine the actual false‐positive and false‐negative rates for tumor cell classification, we used single‐cell data from healthy individuals in the GSE162616 data set and tumor cell lines in the GSE140440 data set for comparative testing. TCfinder exhibits lower false positives and false negatives compared with existing methods (Figures 1C and S3).

## 3 PERFORMANCE OF TCFINDER IN THE SIMULATED DATA SETS

To further demonstrate the robustness and applicability of our method, we validate the model performance by randomly retaining different numbers of genes or randomly inactivating different proportions of pathways (Figure S4A). TCfinder also shows robust performance and significantly outperformed other methods while retaining different number of genes (Figure S4B). This result demonstrates the robust performance of gene pathway expression‐based methods compared to marker gene‐based methods. For simulating different proportions of pathway inactivation, the model's F1 score remained above 0.8 when 60% of the pathways were randomly inactivated (Figure S4C). In addition, models retaining the top four contributing pathways (see next section for details) show significantly higher performance compared with models that do not contain these four pathways (Figure S4D). This further illustrates the important role of these four pathways in TCfinder.

## 4 IDENTIFY PATHWAYS IMPORTANT FOR TUMOR VERSUS NORMAL CELL CLASSIFICATION

Using the limma package to perform differential analysis between the pathway scores of tumor and normal cells, pathways with |log2FoldChange| >1 and false discovery rate <0.05 were shown (Figure 1D). The results indicated that pathways active in tumor cells were primarily related to metabolism, such as oxidative phosphorylation, reactive oxygen species, glutathione metabolism, and glycolysis/gluconeogenesis. The Warburg effect has long established that glycolysis/gluconeogenesis is more active in tumors [17]. Conversely, pathways suppressed in tumor cells were mainly related to immune functions, including antigen processing and presentation, Th1 and Th2 cell differentiation, and natural killer cell‐mediated cytotoxicity. These findings illustrate that, from a single‐cell perspective, tumor cells exhibit a dual characteristic of metabolic hyperactivity and immune suppression.

To further investigate which pathways contribute the most to the discrimination of tumor cells using the GSE148673 data set, we randomly shuffled each pathway and calculated the difference in its loss, which reflects the importance of the pathway. After 100 randomizations, we found the four most important pathways contribute to tumor cell identification: type I diabetes mellitus, oxidative phosphorylation, viral myocarditis, and antigen processing and presentation (Figure 1E). Their pathway scores present strong differential patterns between tumor and normal cells in single‐cell data sets (GSE148673). To verify if these pathways were also prominent in bulk tumors, we collected The Cancer Genome Atlas (TCGA) data. Intriguingly, except for the oxidative phosphorylation pathway, the other three pathways that are related to autoimmunity and antigen presentation, do not present higher pathway scores in tumor samples, indicating that bulk expression cannot reveal the underlying tumor cell expression differences (Figure S5). We further examined the shared antigen presentation (AP)‐related genes in these contributing pathways, which consist of genes including MHC I and MHC II (Figure S6). These AP‐related genes emerged in single‐cell tumor cells, rather than bulk tissue (Figure S7), suggesting that some critical tumor cell‐related information is obscured in bulk tissue RNA‐Seq.

## 5 DISCUSSION

We developed TCfinder, a new gene pathway expression score‐based deep learning method to accurately and rapidly discriminate cancer cells and normal cells in scRNA‐seq. We distinguish cancer cells from normal cells from the perspective of gene pathways and carry out a unique score for each gene pathway so that when only a small number of genes are detected in single cell, the score value will reflect the activity of the entire pathway, which overcomes the sparsity problem of single‐cell data. Based on multiple independent data sets and simulation data, TCfinder shows improved performance than existing methods.

In traditional RNA‐Seq for bulk tissue, the expression profile of tumor cells cannot be accurately determined due to the existence of a large number of cell types in bulk cancer tissue. In scRNA‐Seq, TCfinder identified the oxidative phosphorylation and antigen presentation pathways as the important contributors for tumor versus normal cell discrimination, and tumor cells have higher oxidative phosphorylation and lower antigen presentation gene expression compared with normal cells. Downregulation of antigen presentation pathway has been reported to contribute to tumor immune escape and immunotherapy nonresponsiveness [18, 19]. Interestingly, this difference in single cells is not fully recapitulated in bulk tissues, in TCGA bulk tissues, tumor tissues do not have decreased antigen presentation pathway gene expression compared with surrounding normal tissues. It may be due to the fact that normal cells in bulk tissue mask the true expression of tumor cells. By uncovering potentially hidden information in bulk tumor tissues, TCfinder can be used in clinical settings to identify tumor cells and tailor personalized treatment plans based on the clinical characteristics of these tumor cells.

Although TCfinder has demonstrated its ability to overcome data sparsity in multiple single‐cell data sets and has shown promising performance, it still has some limitations. One of the main limitations is the small number of annotated single‐cell data sets used for training the model. Many cancer tissue‐related single‐cell studies have been reported. However, few data sets have professionally annotated cancer versus normal cell status information. In general, despite its limitations, TCfinder represents a significant improvement over existing methods in addressing the issue of data sparsity in single‐cell annotation. Its success in this regard may also provide useful insights for annotating other cell types.

## 6 CONCLUSIONS

TCfinder is the first tool to distinguish tumor cells from normal cells in single‐cell data from the perspective of gene pathway expression quantification. TCfinder performs well in single‐cell data sets prepared using both 10× and SMART‐Seq2, with prediction accuracy exceeding 0.95. Interestingly, the antigen presentation pathway was identified as the key pathway that distinguishes tumor cells from normal cells, and this antigen presentation pathway expression difference is not recapitulated in bulk tissue RNA‐seq, suggesting that traditional bulk tissue RNA sequencing conceals the true information of a large number of cell states.

## AUTHOR CONTRIBUTIONS

**Chenxu Wu**: Writing—original draft; writing—review and editing; visualization; validation; methodology; software; formal analysis; project administration; data curation. **Wei Ning**: Investigation; methodology; validation; visualization; software. **Tao Wu**: Investigation; writing—review and editing. **Jing Chen**: Data curation. **Huizi Yao**: Writing—review and editing; data curation. **Ziyu Tao**: Data curation. **Xiangyu Zhao**: Data curation. **Kaixuan Diao**: Data curation. **Jinyu Wang**: Data curation. **Weiliang Wang**: Supervision. **Xinxing Li**: Supervision; writing—review and editing; project administration. **Qianqian Song**: Writing—review and editing. **Xue‐Song Liu**: Writing—review and editing; conceptualization; data curation; supervision; funding acquisition; resources; project administration.

## CONFLICT OF INTEREST STATEMENT

The authors declare no conflict of interest.

## ETHICS STATEMENT

No animals or humans were involved in this study.

## Supporting information


**Figure S1:** Distribution of the detected gene numbers in tumor and normal cells. Normal cells present fewer detected genes than tumor cells.
**Figure S2:** Performance comparison between TCfinder and other known methods.
**Figure S3:** TCfinder correctly recognizes most cells in the GSE140440 (tumor cell line) dataset as tumor cells.
**Figure S4:** Performance of TCfinder in the simulated datasets.
**Figure S5:** Pathway scores at bulk tissue and single‐cell level.
**Figure S6:** Identify gene pathways important for tumor vs normal cell classification.
**Figure S7:** Antigen presentation gene expression in bulk tissues.
**Figure S8:** Heatmap showing the performance of TCfinder classifier.
**Figure S9:** Performance comparisons of different machine learning models.
**Figure S10:** Performance of different tumor vs normal cells classification methods for cells with the indicated number of genes.
**Figure S11:** Application of TCfinder in exploring the trajectories/fates of tumor cells.

**Table S1:** Pathways retained for model building after screening.
**Table S2:** List of single cell datasets used in the paper, along with basic statistics
**Table S3:** The performance of different methods.

## Data Availability

Only publicly available data were used in this study, and data sources and handling of these data are described in the Materials and Methods and in Table S2. TCfinder is freely available as an R package at: https://github.com/XSLiuLab/TCfinder. All codes required to reproduce the results reported in this manuscript are freely available at: https://github.com/XSLiuLab/TCfinder/tree/master/inst/analysis. Supplementary materials (results, methods, figures, tables, graphical abstract, slides, videos, Chinese translated version, and updated materials) may be found in the online DOI or iMetaOmics, http://www.imeta.science/imetaomics/.
