# Supplementary material for: TCfinder: Robust tumor cell discrimination in scRNA‐seq based on gene pathway activity
Source: IMetaOmics. 2024 Aug 7;1(1):e22. doi: 10.1002/imo2.22 (PMC12806553; doi:10.1002/imo2.22)
Supplement: Supplementary file 1 — Figure S1: Distribution of the detected gene numbers in tumor and normal cells. Normal cells present fewer detected genes than tumor cells. Figure S2: Performance comparison between TCfinder and other known methods. Figure S3: TCfinder correctly recognizes most cells in the GSE140440 (tumor cell line) dataset as tumor cells. Figure S4: Performance of TCfinder in the simulated datasets. Figure S5: Pathway scores at bulk tissue and single‐cell level. Figure S6: Identify gene pathways important for tumor vs normal cell classification. Figure S7: Antigen presentation gene expression in bulk tissues. Figure S8: Heatmap showing the performance of TCfinder classifier. Figure S9: Performance comparisons of different machine learning models. Figure S10: Performance of different tumor vs normal cells classification methods for cells with the indicated number of genes. Figure S11: Application of TCfinder in exploring the trajectories/fates of tumor cells. [file IMO2-1-e22-s002.docx]

# Supporting information to

# TCfinder: Robust tumor cell discrimination in scRNA-seq based on gene pathway activity

## Running title: Tumor cell discrimination in scRNA-seq

Chenxu Wu^1,2,3#^, Wei Ning^4#^, Tao Wu^1^, Jing Chen^1^, Huizi Yao^1^, Ziyu Tao^1^, Xiangyu Zhao^1^, Kaixuan Diao^1^, Jinyu Wang^1^, Weiliang Wang^5^, Xinxing Li^6^, Qianqian Song^7,8^, Xue-Song Liu^1,9^*

^1^ School of Life Science and Technology, ShanghaiTech University, Shanghai 201210, China;

^2^Shanghai Institute of Biochemistry and Cell Biology, Chinese Academy of Sciences, Shanghai 200032, China;

^3^University of Chinese Academy of Sciences, Beijing 100049, China;

^4^Military Medical Innovation Center, Air Force Medical University, Xi'an 710032, China.

^5^Department of Dermatology, Yangjiang People's Hospital affiliated to Guangdong Medical University, Yangjiang 529599, China.

^6^Department of Colorectal Surgery, Fudan University Shanghai Cancer Center, Shanghai 200032, China;

^7^Center for Cancer Genomics and Precision Oncology, Wake Forest Baptist Comprehensive Cancer Center, Atrium Health Wake Forest Baptist, Winston Salem, NC 27157, USA;

^8^Department of Cancer Biology, Wake Forest School of Medicine, Winston Salem, NC 27101, USA;

^9^Shanghai Clinical Research and Trial Center, Shanghai 201203, China;

^#^ These authors contributed equally: Chenxu Wu, Wei Ning

* Correspondence: [liuxs@shanghaitech.edu.cn](mailto:liuxs@shanghaitech.edu.cn) (Xue-Song Liu)

## SUPPLEMENTARY RESULT

### Performance comparisons of different machine learning models

In addition to the DNN used in TCfinder, we also evaluated four commonly used machine learning framework: LR (Logistic regression), RF (Random forest), SVM (Support vector machines), and XGBOOST (eXtreme gradient boosting) in TCfinder, based on evaluation metrics including F1 score, accuracy, recall, and precision. Specifically, TCfinder presented the best performance when using the DNN model (F1 = 0.97), which was higher than LR (F1 = 0.89), RF (F1 = 0.90), SVM (F1 = 0.94) and XGBOOST (F1 = 0.94) (Figure S8, S9). Noteworthy, TCfinder with DNN model showed excellent perofmrance than others in GSE131309 dataset. This might be due to the data obtained using SMART-Seq2 library preparation, and the generalization ability of other model architectures to different sequencing platforms was poorer than that of the DNN model architecture.

### Comparing TCfinder with existing cell annotation methods

We further explored the performance of these methods in single cell samples with different gene numbers. The results show that TCfinder is significantly better than the other three methods in samples with different gene numbers (Figure S10). Interestingly, we found that the precision of the other three methods is excellent, but the recall is poor. Therefore, we calculated the proportion of tumor cells and normal cells with different gene numbers (Figure S10). The results show that when the number of detected genes is less than 1000, normal cells account for the majority, and when the number of detected genes is more than 2000, tumor cells accounted for the majority. This also confirms that these methods tend to predict cells as normal cells, with a high false negative. At the same time, we find that when the number of detected genes is greater than 2000, the F1 score of TCfinder begins to decline (Figure S10), which may be caused by the mixing of two or more cells during single cell sequencing.

### Application of TCfinder in cancer research

We used anaplastic thyroid cancer samples from the GSE148673 dataset to analyze the trajectory of tumor cells predicted by TCfinder and found that the tumor cells were divided into two clusters (Figure S11). Comparing the pathways of the two different tumor cell clusters show that the three immune-related pathways are not significantly different in the two tumor cell clusters. In oxidative phosphorylation pathway scores, cluster 2 is more similar to normal cells and significantly different from cluster 1. By studying the scores for all pathways in both clusters and normal cells (Figure S11), it is shown that cluster 2 and cluster 1 are at different stages of tumor cell evolution. This analysis demonstrates one example of TCfinder’s application in cancer research. TCfinder can also be applied in tumor cell evolution, classification, targeting related study in scRNA-seq.

## SUPPLEMENTARY METHODS

### Data collection and processing

We collected professionally annotated single-cell datasets from the GEO database (GSE148673, GSE131928, GSE151530, GSE146771, GSE1313309) [1−5], the Neuroblastoma Cell Atlas database (neuroblastoma, Great Ormond Street Hospital, GOSH) [6], and the TISCH2 [7] database (GSE143423, GSE168652, GSE117988, GSE159115, GSE139448, GSE139829, GSE118056, GSE138709) [8−14], obtaining their annotation information and expression matrix for each cell. The GSE148673, GSE131928, GSE143423, GSE168652, GSE117988, and GSE159115 datasets contain a variety of cancer types and a large number of cells, which is used to train the TCfinder model, and several other datasets are used as validation data sets to evaluate the performance of TCfinder. Due to the lack of tumor vs normal cell status professionally annotated datasets, the healthy individuals in the GSE162616 [15] dataset is used as normal cells, and the tumor cell line dataset GSE140440 [16] data is used as tumor cells to test TCfinder. Except for the GSE131309 data set, which is based on SMART-Seq2 method, the other datasets are derived from 10X platform. To make different data comparable, equation 1 is used to standardize the expression matrix of each gene.

$$\begin{aligned} Normalize({Gene}_{i})=\frac{\mathrm{Gen}e_{i}}{\sum_{j=1}^{m} \mathrm{Gen}e_{j}}*{10}^{4}\#\left( 1 \right) \end{aligned}$$

Gene is the count value, and m is the number of all genes in single cell.

### Calculate pathway scores

All 335 human pathways and their corresponding genes were obtained from the KEGG (Kyoto Encyclopedia of Genes and Genomes) database. Although single-cell data are relatively sparse (containing many data with counts of 0), data with counts of 0 is also potentially meaningful, so all data are used to calculate pathway scores. To make the pathway scores as representative of the true cellular state as possible, we scored each pathway using equation 2.

$$\begin{aligned} PathyScore=\frac{\sum_{i=1}^{n} \log_{2} \left( Normalize_{n}+1 \right)}{n}\#\left( 2 \right) \end{aligned}$$

n is the number of genes for each pathway and Normalize is the result of Gene standardization.

### Feature pathway screening

In order to screen out the pathways that can represent tumor and normal cells, we used training dataset to analyze the difference in the scores of tumor and normal cell pathways through the Wilcoxon test, and select the pathways that are significantly different between tumor and normal cells (*p* < 0.05). We finally obtained 213 pathways for subsequent model construction.

### Model construction

The DNN architecture is used as the basis to build the classification model and the 213 pathway scores identified above as input. The training set is divided into two data sets in a ratio of 8:2, with Gridsearch [17] used for cross-validation and hyperparameter tuning. Specifically, the optimal number of layers is selected from 3 to 6. The dimension of first and last layer is selected among the range from 200 to 500, and 10-30, respectivley. The dropout rates are 0.1, 0.3, and 0.5.

The number of nodes gradually decreases from the first hidden layer to the last hidden layer, and the reduced step formula is:

$$\begin{aligned} \begin{aligned} reduced step = \frac{first_{\_}layer\_nodes-last_{\_}layer\_nodes}{n\_layers-1}\# \end{aligned}\#\left( 3 \right) \end{aligned}$$

First_layer_nodes is the number of nodes in the first hidden layer, last_ layer_nodes is the number of nodes in the last hidden layer, n_layers is the number of hidden layers. The dropout rate of each hidden layer is also different. Similarly, given the dropout rate of the first layer, the dropout rate of the subsequent hidden layer continues to decrease until it is 0.5%. The reduced step size is:

$$\begin{aligned} \begin{aligned} reduced step = round\left( \frac{dropout}{n\_layer-1},2 \right)\#\# \end{aligned}\#\left( 4 \right) \end{aligned}$$

Dropout is the proportion of randomly deleted nodes in the first hidden layer.

### Model framework comparison

In order to highlight the advantages of our model architecture, under the same training set, the LR [18], RF [19], SVM [20], and XGBOOST are also used as model architectures for model training and testing. Among them, the parameters of the LR model are max_iter is 1000 and n_jobs is 1.The model parameters of RF are 200 for n_estimators and 1234 for random_state.SVM model parameters are the default parameters.The parameters of the XGBOOST model are min_child_weight is 6, max_depth is 15, and objective is binary:logitraw.

### Comparison of different methods

We compared our own method with existing methods that discriminate tumor cells from normal cells. Among them, the characteristic gene set reserved for themselves by the ikarus method, and the marker gene set required by the SCINA and scMRMA methods are obtained from the CancerSCEM database [21]. The copykat runtime parameters are set according to the recommendations. Due to the large number of marker genes required by other methods, for the undetected marker genes, in order to predict the normal operation of the program, we first assign them to 0, and then make predictions, and compare them through model evaluation indicators such as F1 score, precision, recall, Accuaacy, etc.

### Calculate the importance of the pathway

In order to determine which pathways have a greater impact on model performance, the scores of each pathway are artificially changed. In the GSE148673 dataset pathway scores, randomly disrupted the scores of 213 pathways one by one and then obtained the predicted loss and accuracy after the scores of each pathway were disrupted. This random process was repeated 100 times, and the average value of the corresponding loss of each pathway was used as a reference for the importance of the pathway.

### Random gene inactivation simulation

In the GSE148673 dataset, we first count the number of genes with a count value greater than 0 in each cell, and according to the distribution of the number of genes with a count greater than 0 in the statistics. For each cell, we choose to randomly retain 500, 1,000, 1,500, 2,000, and 2,500 genes with count values greater than 0. For cells that have not measured the target number, we choose to delete them directly. We performed 100 random simulations of each condition, using TCfinder, ikarus, SCINA, scMRMA and other methods to predict the cells after the simulated inactivation of genes, and compare the effects and stability of different methods.

### Random pathway inactivation simulation

In order to verify the robustness of TCfinder, we performed pathway simulation inactivation by randomly deleting 5%、10%、20%、30%、40%、50%、60%、70%、80%、90%. The pathway score of each inactivated pathway was simulated 100 times, and then TCfinder was used to make predictions and compare the relationship between the number of inactivated pathways and the performance of the model.

### Gene Set Enrichment Analysis (GSEA) analysis

Breast cancer samples and their corresponding normal samples were collected in TCGA, used R package DESeq2 (version 1.34) [22] to perform differential analysis on the raw counts value, and used Equation 5 to calculate the rank value for the differential analysis results. The rank value was used as the GSEA [23] input data, and the hsa00190, hsa04612, hsa04940, and hsa05416 pathways in KEGG were used as the gene set to calculate the enrichment score. GSEA analysis was done using the R package GSEABase (version 1.42) [24].

$$\begin{aligned} rank=-\frac{log2FoldChange}{\left| log2FoldChange \right|}*log10\left( \mathrm{padj} \right)\#\left( 5 \right) \end{aligned}$$

log2(FoldChange) is the fold difference of the samples, padj refers to the corrected *p*-value.

### Gene Set Variation Analysis (GSVA) analysis

Normalized enrichment score (NES) of pathways and functional annotation was calculated by the gene set variation analysis (GSVA) method using the R package GSVA (version 1.42) [25]. Genes of the hsa00190, hsa04612, hsa04940, and hsa05416 pathways were downloaded from the KEGG database to run GSVA enrichment analysis. The samples analyzed were breast cancer samples and their corresponding normal samples from the TCGA database.

### Trajectory analysis

For tumor evolution, trajectory analysis was performed using the R package Monocle3 (version 1.2.9) [26] to determine the evolutionary relationships between different cancer cell clusters. Default parameters were used in Monocle3, with the parameter “num_dim” = 50, for the anaplastic thyroid cancer scRNA-seq dataset (accession code: GSE148673).

### Statistical analysis

All statistical tests and visualization analyses were performed with R. P values showed in boxplot were calculated by Wilcoxon tests. The following convention of symbols indicated statistical signifcance: ns: *p* > 0.05, *: *p* ≤ 0.05, **: *p* ≤ 0.01, ***: *p* ≤ 0.001, ****: *p* ≤ 0.0001.

## Software

TCfinder requires only one CPU for predicting tumor cells. Using an Intel(R) Xeon(R) CPU E5-2620 v2 @ 2.10GHz processor, predicting 100,000 cells takes only 7.6 seconds, with a memory usage of 514MB during the process. The tutorial for using TCfinder, along with the detailed training process and parameters, can be found at the following link.

https://github.com/XSLiuLab/TCfinder/tree/master/inst/analysis

## REFERENCES

1. Gao, Ruli, Shanshan Bai, Ying C. Henderson, Yiyun Lin, Aislyn Schalck, Yun Yan, Tapsi Kumar, et al. 2021. “Delineating copy number and clonal substructure in human tumors from single-cell transcriptomes.” *Nature biotechnology* 39: 599-608. <https://doi.org/10.1038/s41587-020-00795-2>.

2. Ma, Lichun, Limin Wang, Subreen A Khatib, Ching-Wen Chang, Sophia Heinrich, Dana A Dominguez, Marshonna Forgues, et al. 2021. “Single-cell atlas of tumor cell evolution in response to therapy in hepatocellular carcinoma and intrahepatic cholangiocarcinoma.” *Journal of Hepatology* 75: 1397-1408. <https://doi.org/10.1016/j.jhep.2021.06.028>.

3. Zhang, Lei, Ziyi Li, Katarzyna M Skrzypczynska, Qiao Fang, Wei Zhang, Sarah A O’Brien, Yao He, et al. 2020. “Single-cell analyses inform mechanisms of myeloid-targeted therapies in colon cancer.” *cell* 181: 442-459. e429. <https://doi.org/10.1016/j.cell.2020.03.048>.

4. Jerby-Arnon, Livnat, Cyril Neftel, Marni E Shore, Hannah R Weisman, Nathan D Mathewson, Matthew J McBride, Brian Haas, et al. 2021. “Opposing immune and genetic mechanisms shape oncogenic programs in synovial sarcoma.” *Nature medicine* 27: 289-300. <https://doi.org/10.1038/s41591-020-01212-6>.

5. Neftel, Cyril, Julie Laffy, Mariella G Filbin, Toshiro Hara, Marni E Shore, Gilbert J Rahme, Alyssa R Richman, et al. 2019. “An integrative model of cellular states, plasticity, and genetics for glioblastoma.” *cell* 178: 835-849. e821. <https://doi.org/10.1016/j.cell.2019.06.024>.

6. Kildisiute, Gerda, Waleed M Kholosy, Matthew D Young, Kenny Roberts, Rasa Elmentaite, Sander R van Hooff, Clarissa N Pacyna, et al. 2021. “Tumor to normal single-cell mRNA comparisons reveal a pan-neuroblastoma cancer cell.” *Science advances* 7: eabd3311. <https://doi.org/10.1126/sciadv.abd3311>.

7. Han, Ya, Yuting Wang, Xin Dong, Dongqing Sun, Zhaoyang Liu, Jiali Yue, Haiyun Wang, Taiwen Li, Chenfei Wang. 2023. “TISCH2: expanded datasets and new tools for single-cell transcriptome analyses of the tumor microenvironment.” *Nucleic acids research* 51: D1425-D1431. <https://doi.org/10.1093/nar/gkac959>.

8. Wang, Liang, Jinxiang Dai, Run-Run Han, Lei Dong, Dayun Feng, Gang Zhu, Wei Guo, et al. 2019. “Single-cell map of diverse immune phenotypes in the metastatic brain tumor microenvironment of non small cell lung cancer.” *bioRxiv* 2019.2012. 2030.890517. <https://doi.org/10.1101/2019.12.30.890517>.

9. Li, Chunbo, Luopei Guo, Shengli Li, Keqin Hua. 2021. “Single-cell transcriptomics reveals the landscape of intra-tumoral heterogeneity and transcriptional activities of ECs in CC.” *Molecular Therapy-Nucleic Acids* 24: 682-694. <https://doi.org/10.1016/j.omtn.2021.03.017>.

10. Paulson, KG, V Voillet, MS McAfee, DS Hunter, FD Wagener, M Perdicchio, WJ Valente, et al. 2018. “Acquired cancer resistance to combination immunotherapy from transcriptional loss of class I HLA.” *Nature Communications* 9: 3868. <https://doi.org/10.1038/s41467-018-06300-3>.

11. Zhang, Yuping, Sathiya P Narayanan, Rahul Mannan, Gregory Raskind, Xiaoming Wang, Pankaj Vats, Fengyun Su, et al. 2021. “Single-cell analyses of renal cell cancers reveal insights into tumor microenvironment, cell of origin, and therapy response.” *Proceedings of the National Academy of Sciences* 118: e2103240118. <https://doi.org/10.1073/pnas.2103240118>.

12. Wang, Rong, Roshan Sharma, Xiaojuan Shen, Ashley M Laughney, Kosuke Funato, Philip J Clark, Monika Shpokayte, et al. 2020. “Adult human glioblastomas harbor radial glia-like cells.” *Stem Cell Reports* 14: 338-350. <https://doi.org/10.1016/j.stemcr.2020.01.007>.

13. Durante, Michael A, Daniel A Rodriguez, Stefan Kurtenbach, Jeffim N Kuznetsov, Margaret I Sanchez, Christina L Decatur, Helen Snyder, et al. 2020. “Single-cell analysis reveals new evolutionary complexity in uveal melanoma.” *Nature Communications* 11: 496. <https://doi.org/10.1038/s41467-019-14256-1>.

14. Zhang, Min, Hui Yang, Lingfei Wan, Zhaohai Wang, Haiyang Wang, Chen Ge, Yunhui Liu, et al. 2020. “Single-cell transcriptomic architecture and intercellular crosstalk of human intrahepatic cholangiocarcinoma.” *Journal of Hepatology* 73: 1118-1130. <https://doi.org/10.1016/j.jhep.2020.05.039>.

15. Liu, Huan, Ronghua Zhao, Rongrong Qin, Haoyu Sun, Qiang Huang, Lianxin Liu, Zhigang Tian, et al. 2022. “Panoramic comparison between NK cells in healthy and cancerous liver through single-cell RNA sequencing.” *Cancer Biology & Medicine* 19: 1334. <https://doi.org/10.20892/j.issn.2095-3941.2022.0050>.

16. Schnepp, Patricia M, Greg Shelley, Jinlu Dai, Nicole Wakim, Hui Jiang, Atsushi Mizokami, Evan T Keller. 2020. “Single-cell transcriptomics analysis identifies nuclear protein 1 as a regulator of docetaxel resistance in prostate cancer cells.” *Molecular Cancer Research* 18: 1290-1301. <https://doi.org/10.1158/1541-7786.MCR-20-0051>.

17. Fabian, Pedregosa. 2011. “Scikit-learn: Machine learning in Python.” *Journal of machine learning research 12* 2825. <https://doi.org/10.5555/1953048.2078195>.

18. LaValley, Michael P. 2008. “Logistic regression.” *Circulation* 117: 2395-2399. <https://doi.org/10.1161/CIRCULATIONAHA.106.682658>.

19. Rigatti, Steven J. 2017. “Random forest.” *Journal of Insurance Medicine* 47: 31-39. <https://doi.org/10.17849/insm-47-01-31-39.1>.

20. Guenther, Nick, Matthias Schonlau. 2016. “Support Vector Machines.” *The Stata Journal* 16: 917-937. <https://doi.org/10.1177/1536867X1601600407>.

21. Zeng, Jingyao, Yadong Zhang, Yunfei Shang, Jialin Mai, Shuo Shi, Mingming Lu, Congfan Bu, et al. 2022. “CancerSCEM: a database of single-cell expression map across various human cancers.” *Nucleic acids research* 50: D1147-D1155. <https://doi.org/10.1093/nar/gkab905>.

22. Love, Michael I, Wolfgang Huber, Simon Anders. 2014. “Moderated estimation of fold change and dispersion for RNA-seq data with DESeq2.” *Genome biology* 15: 1-21. <https://doi.org/10.1186/s13059-014-0550-8>.

23. Subramanian, Aravind, Pablo Tamayo, Vamsi K Mootha, Sayan Mukherjee, Benjamin L Ebert, Michael A Gillette, Amanda Paulovich, et al. 2005. “Gene set enrichment analysis: a knowledge-based approach for interpreting genome-wide expression profiles.” *Proceedings of the National Academy of Sciences* 102: 15545-15550. <https://doi.org/10.1073/pnas.0506580102>.

24. Gentleman, MMFS. 2022. “GSEABase: Gene Set Enrichment Data Structures and Methods.” *R package version* 1: <https://doi.org/10.18129/B9.bioc.GSEABase>.

25. Hänzelmann, Sonja, Robert Castelo, Justin Guinney. 2013. “GSVA: gene set variation analysis for microarray and RNA-seq data.” *BMC bioinformatics* 14: 1-15. <https://doi.org/10.1186/1471-2105-14-7>.

26. Qiu, Xiaojie, Andrew Hill, Jonathan Packer, Dejun Lin, Yi-An Ma, Cole Trapnell. 2017. “Single-cell mRNA quantification and differential analysis with Census.” *Nature methods* 14: 309-315. <https://doi.org/10.1038/nmeth.4150>.

## SUPPLEMENTARY FIGURE

**
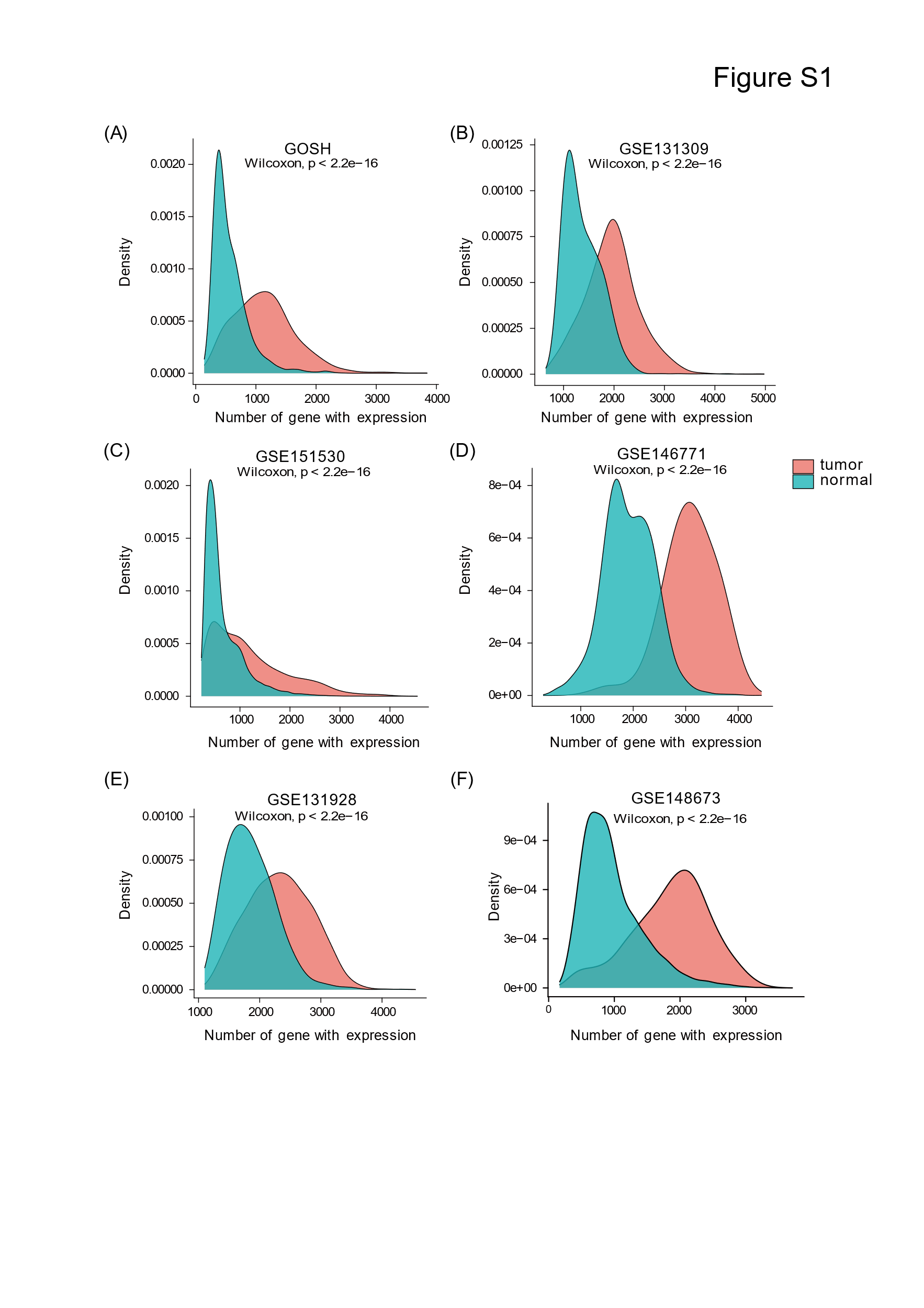
**

**Figure S1 Distribution of the detected gene numbers in tumor and normal cells.** (A-F) Distribution of the detected gene numbers in tumor and normal cells in GOSH (A), GSE131309 (B), GSE151530 (C), GSE146771 (D), GSE131928 (E) and GSE148673 (F) datasets. Normal cells present fewer detected genes than tumor cells.

**
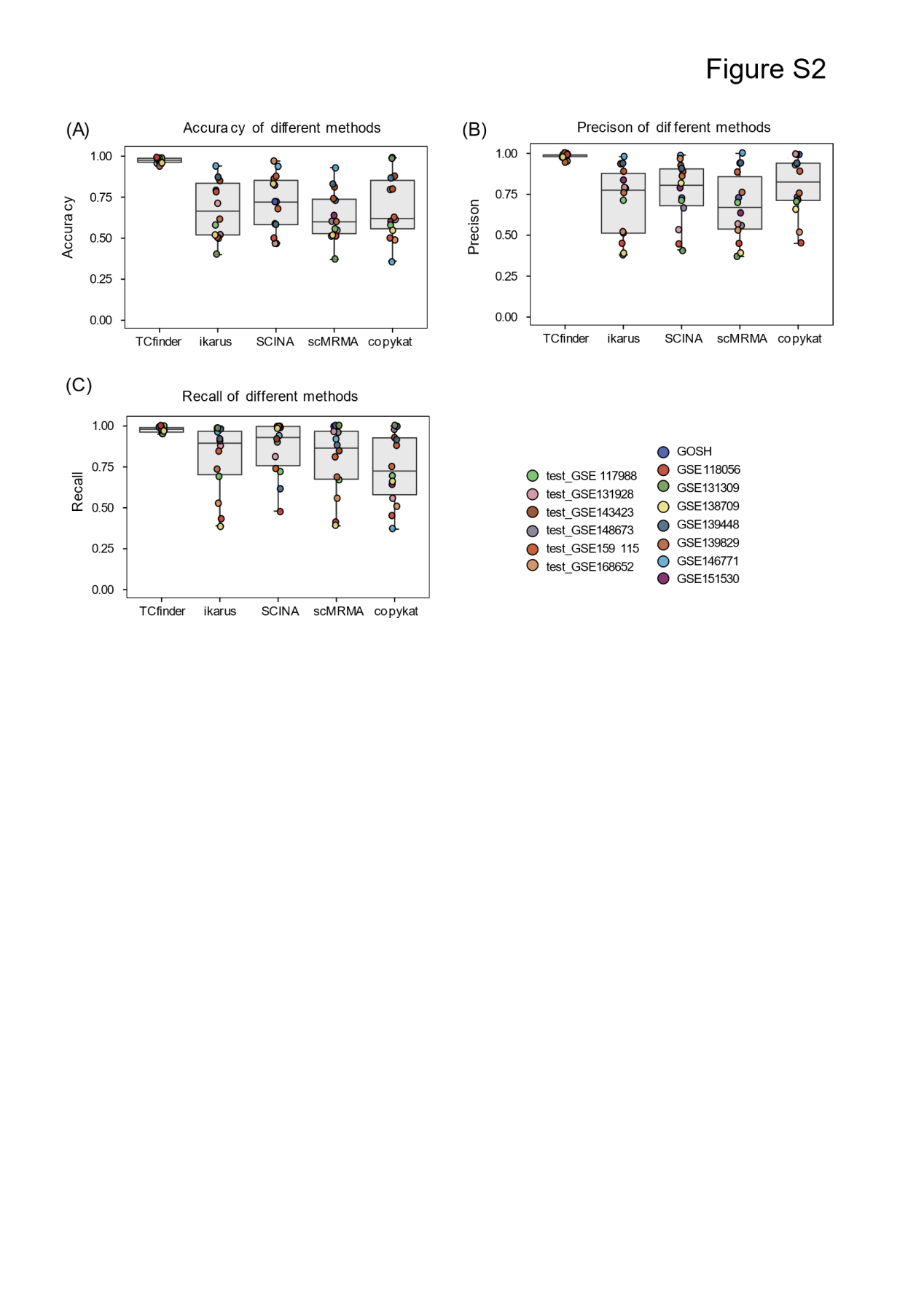
**

**Figure S2 Performance comparison between TCfinder and other known methods.** (A-C) Tumor vs normal cells classification performance of TCfinder and other known methods (ikarus, SCINA, copykat, and scMRMA). Accuracy (A), precision (B) and recall (C) for each methods in independent validation datasets are shown.

**
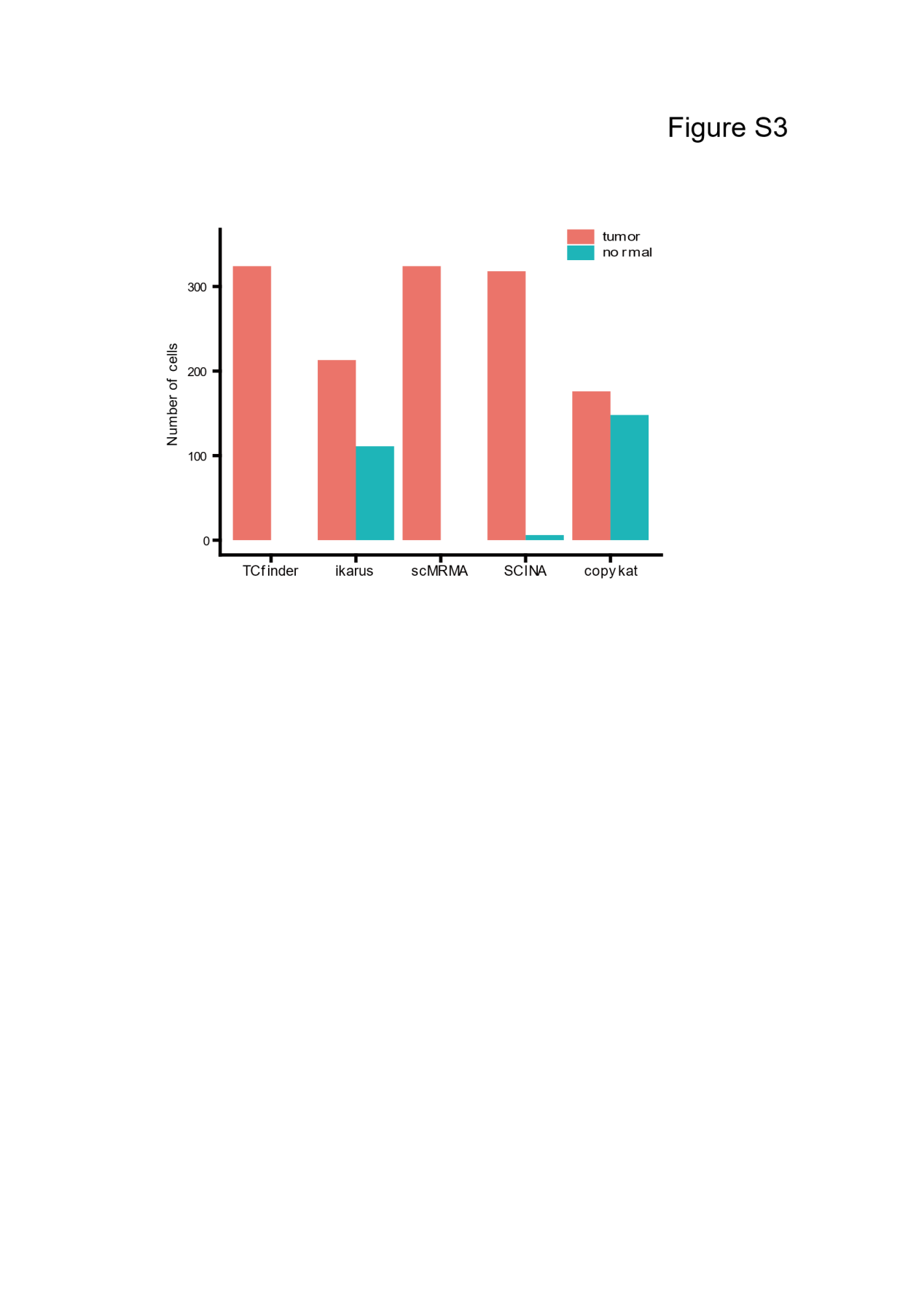
**

**Figure S3 TCfinder correctly recognizes most cells in the GSE140440 (tumor cell line) datase as tumor cells.**

**
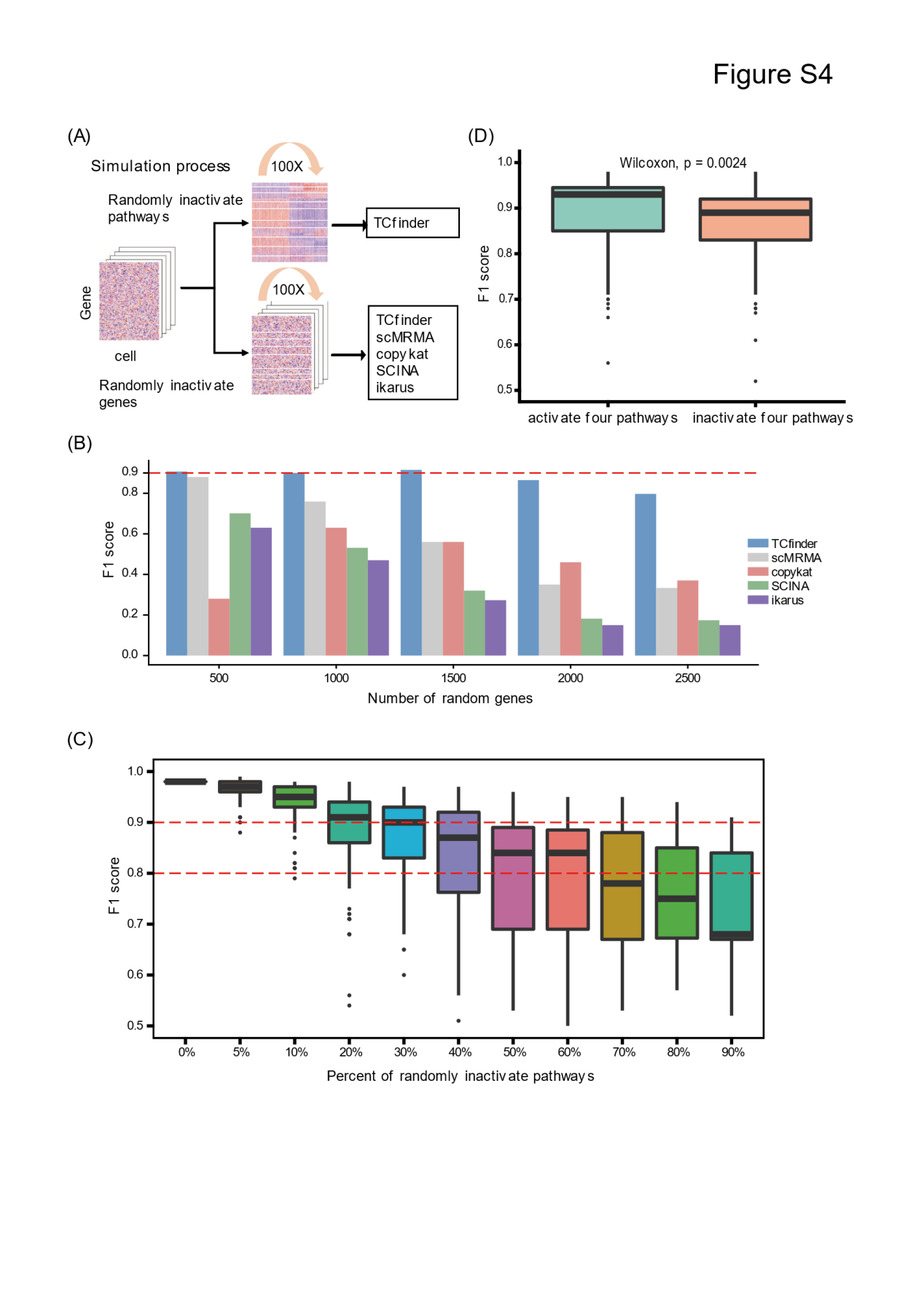
**

**Figure S4** **Performance of TCfinder in the simulated datasets.** (A) Gene and pathway inactivation simulation process. Randomly assign gene and pathway values in cells to zero, and compare the performance of different methods. Repeat the process100 times. (B) Comparing the performance of different methods in classifying tumor and normal cells with the number of indicated random genes. Randomly keep 500, 1000, 1500, 2000 and 2500 genes without inactivation, and compare the F1 score of different methods for classifying tumor cells and normal cells. The average F1 scores of 100 repeats are shown. (C) Performance of TCfinder at the indicated percent of randomly inactivated pathways. For each simulation, F1 score of 100 repeats is shown. (D) Effects of the four most important pathways on the performance of simulated inactivated pathways. Divide the inactivated 20% pathways into whether they contain the four most important pathways, and compare the F1 scores of TCfinder of tumor cells and normal cells. Repeat 100 times.

**
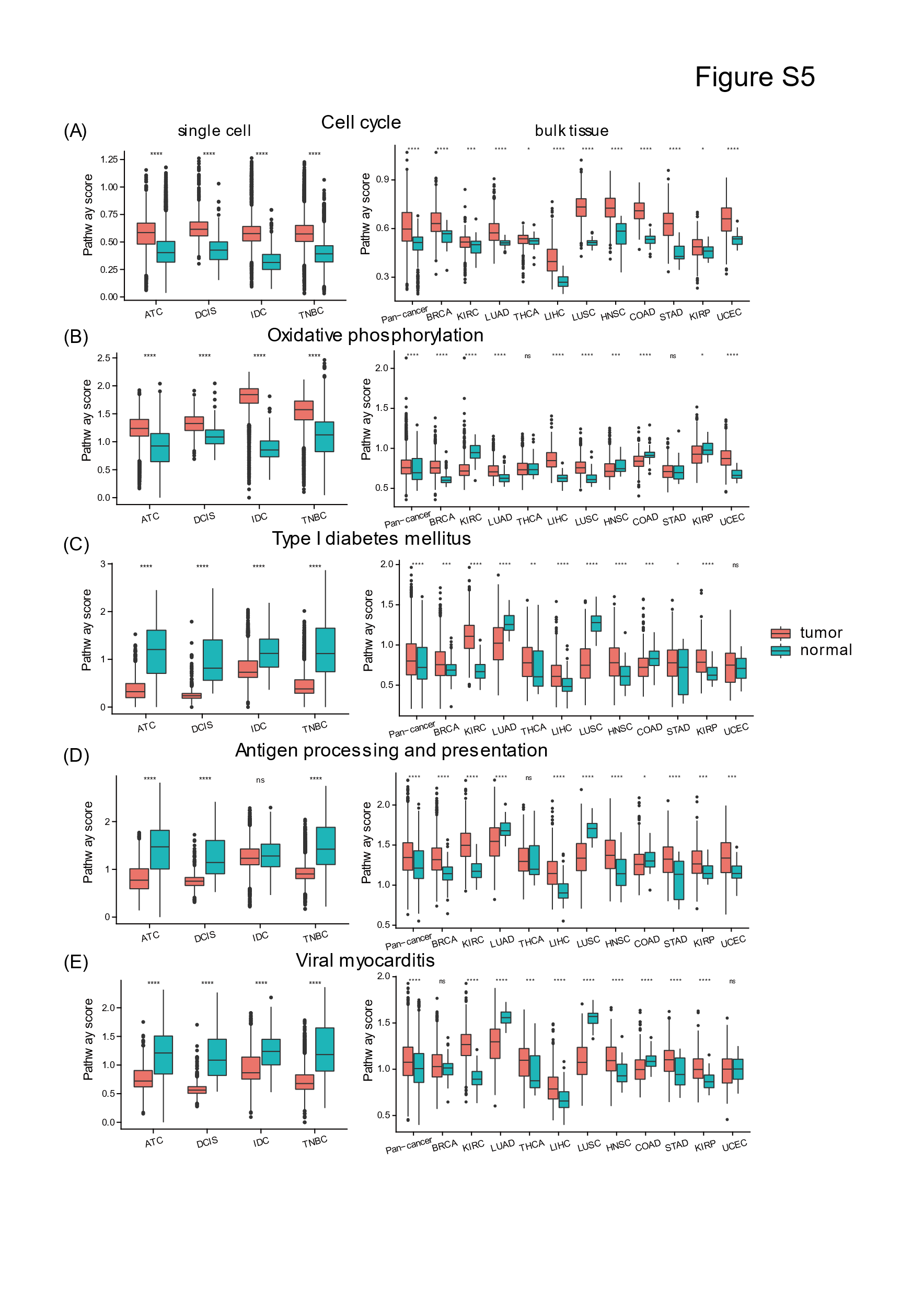
**

**Figure S5 Pathway scores at bulk tissue and single-cell level.** (A-E) Pathway scores of cell cycle (A), oxidative phosphorylation (B), type I diabetes mellitus (C), antigen processing and presentation (D), and viral myocarditis (E) at single cell level and bulk tissue level. The cell cycle pathway is used as an internal reference to measure changes in other pathways. Wilcoxon test P values are shown. ns: *p* > 0.05, *: *p* ≤ 0.05, **: *p* ≤ 0.01, ***: *p* ≤ 0.001, ****: *p* ≤ 0.0001.

**
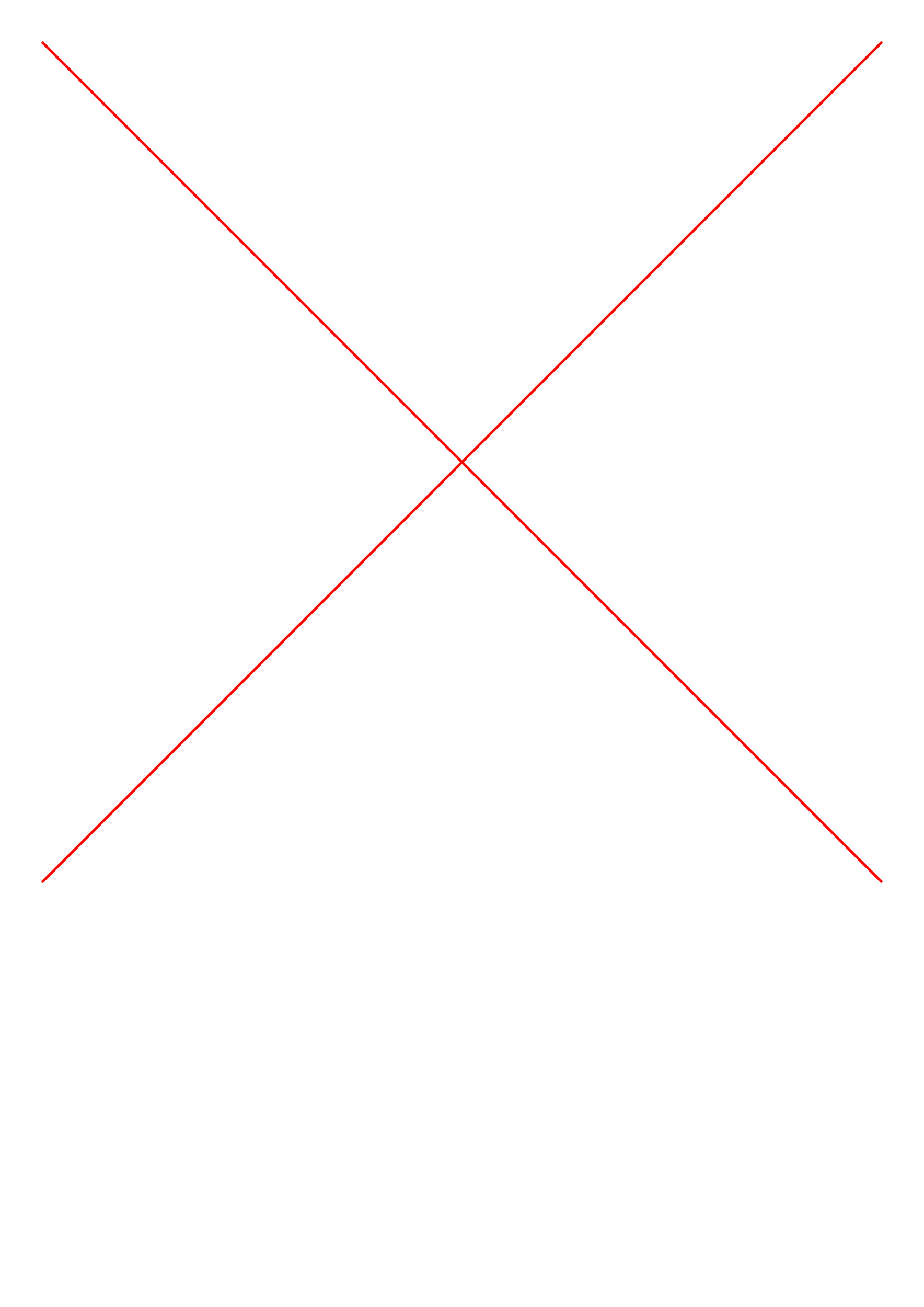
**

**Figure S6** **Identify gene pathways important for tumor vs normal cell classification.** (A) Analysis of genes in the four most important pathways by Venn. The overlapping part is the number of genes shared by different pathways. (B) Antigen-presenting gene expression in tumor and normal cells. In single cells, mRNA expression of MHC class I and class II antigen presentation related genes in tumor and normal cells. Wilcoxon test *P* values are shown. ns: *p* > 0.05, *: *p* ≤ 0.05, **: *p* ≤ 0.01, ***: *p* ≤ 0.001, ****: *p* ≤ 0.0001.

**
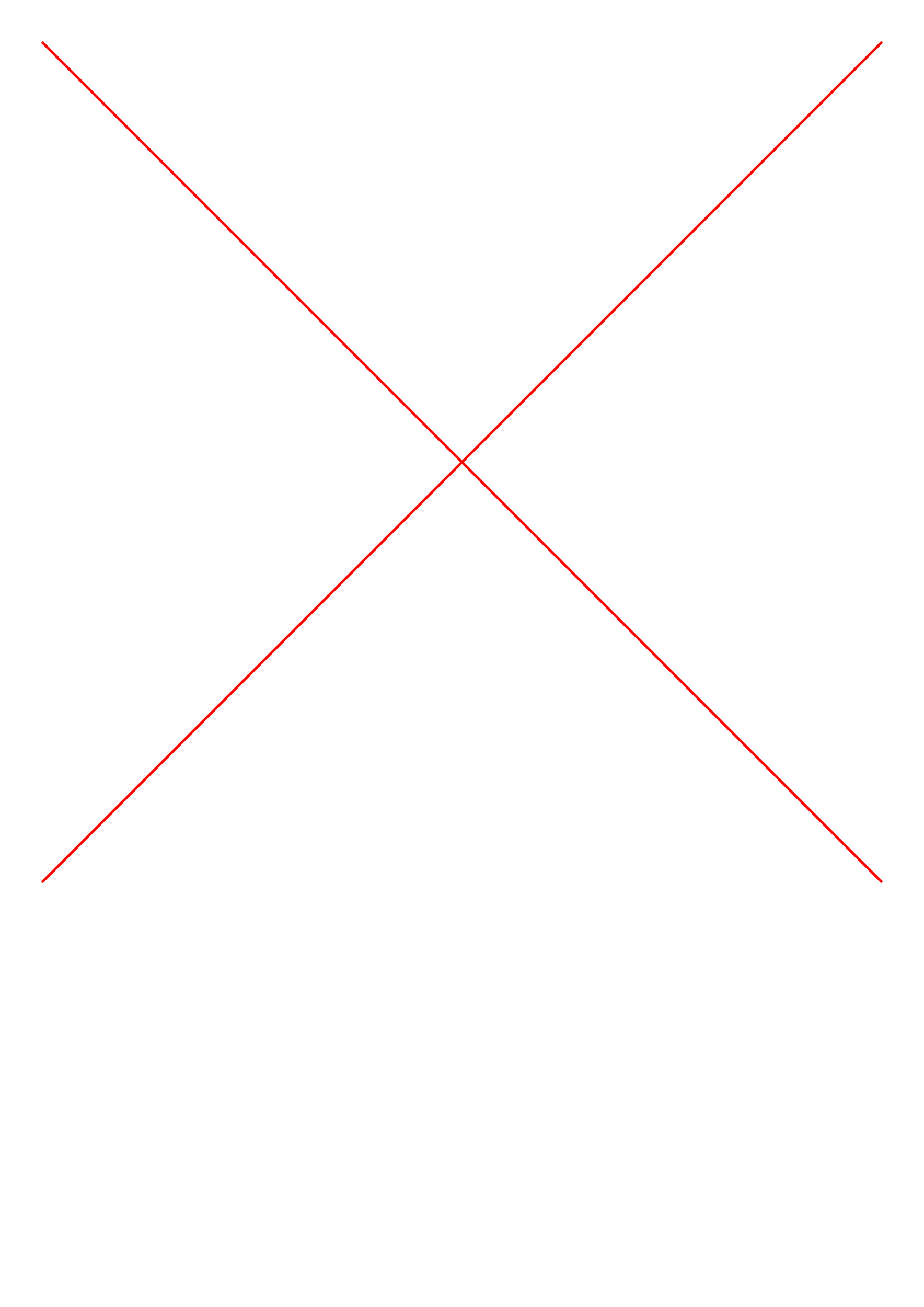
**

**Figure S7 Antigen presentation gene expression in bulk tissues.** (A) Antigen-presenting gene expression in tumor and normal bulk tissue. The gene expression uses the tpm value, and the data is the breast cancer sample of TCGA. Wilcoxon test *P* values are shown. ns: *p* > 0.05, *: *p* ≤ 0.05, **: *p* ≤ 0.01, ***: *p* ≤ 0.001, ****: *p* ≤ 0.0001. (B) GSEA (Gene Set Enrichment Analysis) analysis of 4 important pathways. KEGG pathway acronym: hsa00190 (Oxidative phosphorylation), hsa04612 (Antigen processing and presentation), hsa04940 (Type I diabetes mellitus), hsa05416 (Viral myocarditis). The data is the breast cancer sample of TCGA. (C) GSVA (Gene Set Variation Analysis) analysis of 4 important pathways. The data is the breast cancer sample of TCGA. Wilcoxon test P values are shown. ns: *p* > 0.05, *: *p* ≤ 0.05, **: *p* ≤ 0.01, ***: *p* ≤ 0.001, ****: *p* ≤ 0.0001.

**
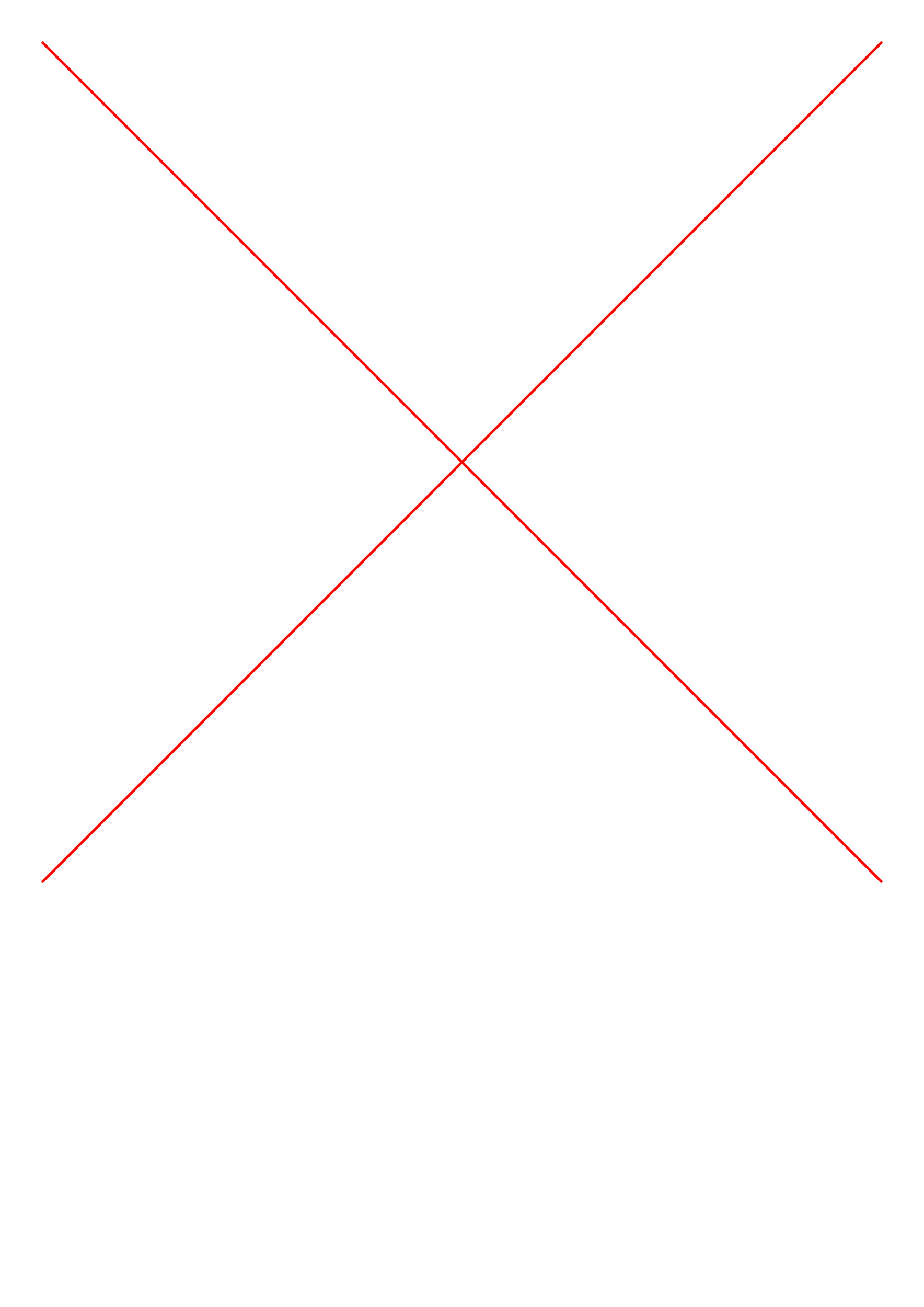
**

**Figure S8 Heatmap showing the performance of TCfinder classifier.** (A) Performance of TCfinder classifier on the GSE131309. (B-E) Prediction results of LR (B), FR (C), SVM (D), and XGBOOST (E) models in GSE131309 dataset.

**
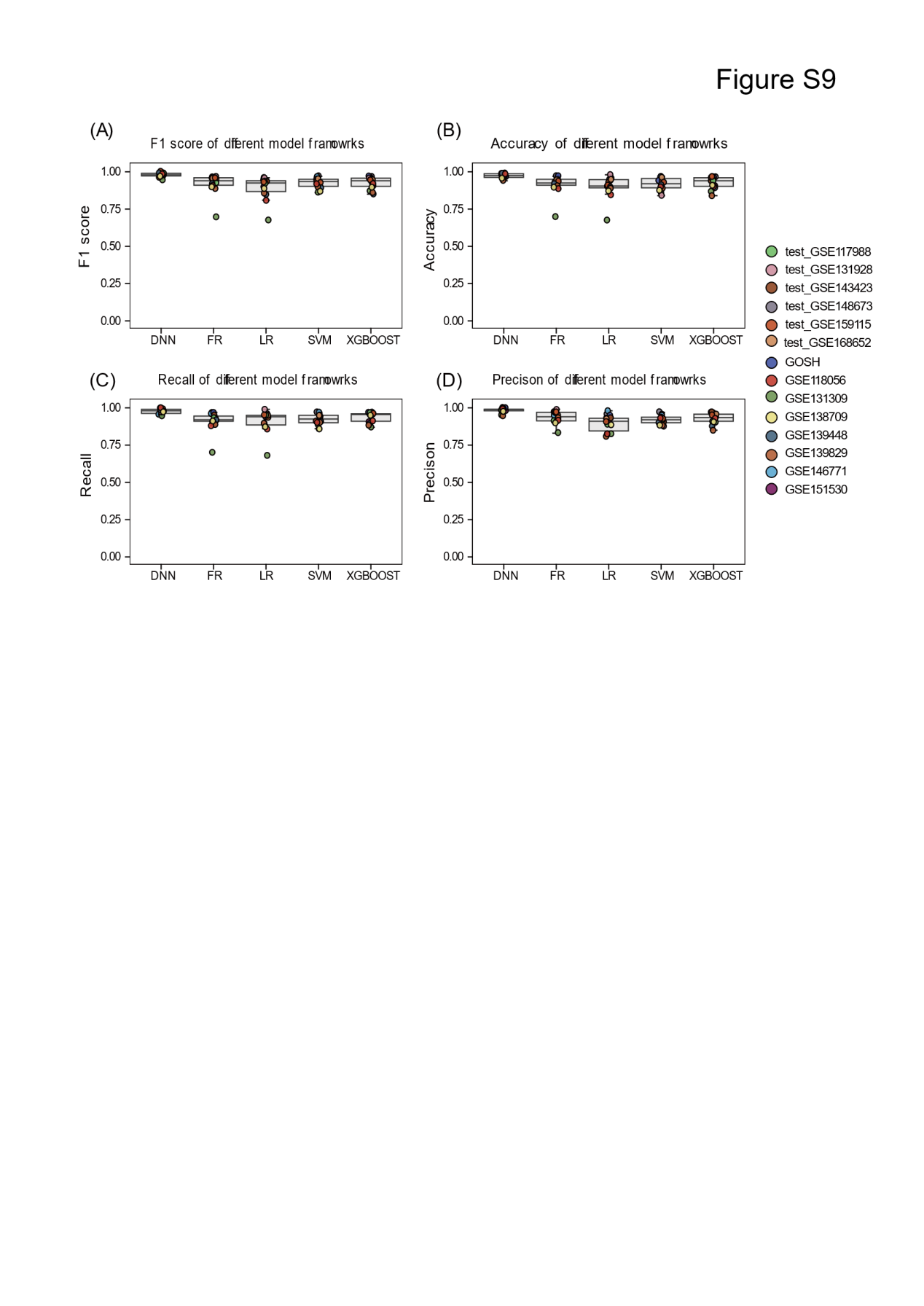
**

**Figure S9 Performance comparisons of different machine learning models.** (A-D) Tumor vs normal cell classification performance of different machine learning frameworks. F1 score (A), accuracy (B), recall (C) and precision (D) for each classification frameworks (DNN, LR, FR, SVM and XGBOOST) are reported.

**
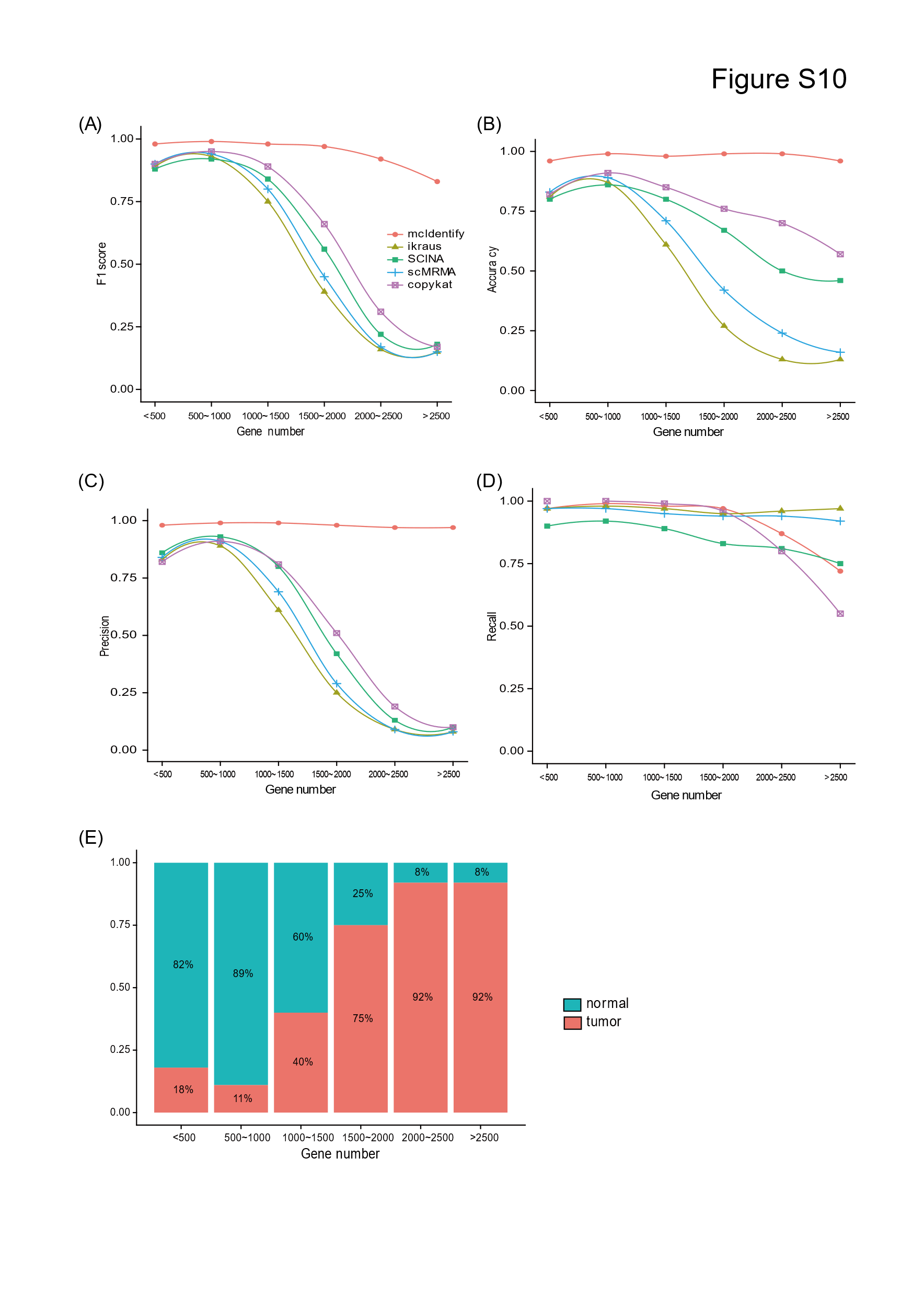
**

**Figure S10 Performance of different tumor vs normal cells classification methods for cells with the indicated number of genes.** (A-D) In cells with different numbers of detected genes, the F1 score (A), accuracy (B), precision (C), and recall (D) of different methods (TCfinder, ikraus, SCINA, copykat and scMRMA) for classifying tumor cells and normal cells from GSE148673 datasets are shown. (E) The proportion of tumor and normal cells with different numbers of detected genes in GSE148673 datasets.

**
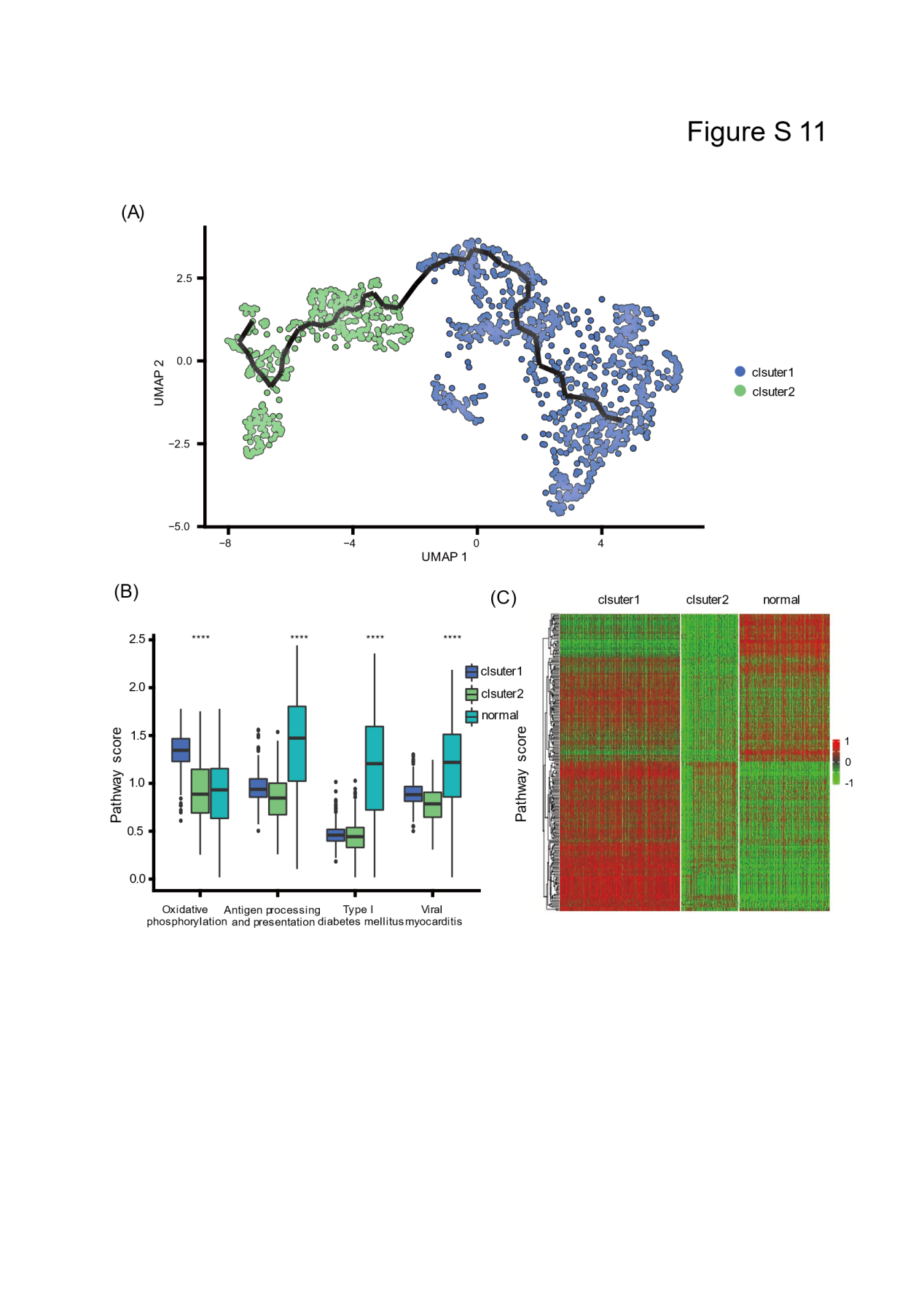
**

**Figure S11 Application of TCfinder in exploring the trajectories/fates of tumor cells.** (A) Trajectory manifold of tumor cells using the Monocle 3 algorithm. Solid line represent distinct tumor cell trajectories/fates defined by expression profiles. Data are tumor cells in anaplastic thyroid cancer identified using TCfinder in GSE148673. (B) Four important pathway scores in tumor cells of different trajectories and normal cells. Wilcoxon test *P* values are shown. ns: *p* > 0.05, *: *p* ≤ 0.05, **: *p* ≤ 0.01, ***: *p* ≤ 0.001, ****: *p* ≤ 0.0001. KEGG pathway acronym: hsa00190 (Oxidative phosphorylation), hsa04612 (Antigen processing and presentation), hsa04940 (Type I diabetes mellitus), hsa05416 (Viral myocarditis). (C) Heatmap of pathway scores in tumor cells of different trajectories and normal cells.
